# Midwifery and Nursing Students’ Communication Skills and Life Orientation: Correlation with Stress Coping Approaches

**Published:** 2013-06-27

**Authors:** Gülsün Özdemir, Hatice Kaya

**Affiliations:** 1 Department of Fundamentals of Nursing, Nursing Faculty, Istanbul University, Istanbul, Turkey

**Keywords:** Nursing and midwifery students, Communication, Optimism, Coping skills

## Abstract

**Background:**

Methods learnt by nursing and midwifery students’ such as communication skills, optimisim and coping with stress would be used in their profeesional life. It is very important to promote their positive thinking and communication skills to raise coping with stress.

**Objectives:**

This cross sectional study was performed to examine the nursing and midwifery students’ communication skills and optimistic life orientation and its correlation with coping strategies with stress.

**Materials and Methods:**

The study population included 2572 students who were studying in departments of nursing and midwifery in Istanbul. The sample was included 1419 students. Three questionnaires including Communication Skills Test, Life Orientation Test and Ways of Coping Inventory were used for data collection. The data were evaluated by calculating frequency, percentage, arithmetic mean, standard deviation and Pearson correlation coefficient.

**Results:**

Students’ total mean score from the Communication Skills Scale was 165.27 ± 15.39 and for the Life Orientation Test was 18.51 ± 4.54. There was a positive correlation between their Life Orientation scores and the scores for self confidence (r = 0.34, P < 0.001), optimistic approach (r = 0.42, P < 0.001), and seeking social help (r = 0.17, P < 0.001). Also there was a significant positive correlation between Communication skill scores and self confidence (r = 0.46, P < 0.001), optimistic (r = 0.37, P < 0.001) and seeking social help approaches (r = 0.29, P < 0.001), but there was a significant negative correlation between communication skill scores and scores for helpless (r = -0.29, P < 0.001) and submissive approaches (r = -0.36, P < 0.001).

**Conclusions:**

As scores of students in optimistic life orientation and communication skills increased self confidence approach, optimistic, and social support seeking scores increased, whereas helpless, and submissive scores decreased.

## 1. Background

It is only possible with a healthy personality development for human being, defined as a bio-physiological, psychological and socio-cultural entity, to accomplish his social role efficiently, to pursue a regular and happy life and to gain a meaning in society. Interaction between individual’s behavioral state or personality traits and stressful life events affects human's attitude toward life. Stress is a process which can lead to anxiety, sadness and collapse. Stress is defined as physical and mental reactions shown to dangers that threaten and compel the individual. On the other hand, coping is cognitive, emotional and behavioral efforts to manage the conflicts among the intrinsic and extrinsic demands which can go beyond individual’s resources and compel them. Coping consists of two parts: problem-focused and emotion-focused. Problem-focused coping is about conscious efforts which include cognitive problem-solving, decision-making, solving disagreements between people, getting advice, determining the goal and making good use of time to change the situation. Emotion-focused coping is about cognitive and behavioral efforts to overcome stressful feelings (1-4).

Of all the suggestions made to cope with stressful events, being optimistic and having a positive behavior have a great role (5, 6). Optimism is to look at events from a positive point of view. Optimist individuals always think that things would turn out positively due to their talents, being very lucky and popular among other people. Thinking that their behaviors would have positive effects on the results, they continue to make efforts for such results (6,7).

According to the literature, optimism is related to active problem-solving approaches; whereas, pessimism is related to approaches toward emotions. Therefore, individuals with high optimism can cope with sources of stress more effectively in life (5, 7-9). In a study where La Montagne et al. (8) analyzed optimism, anxiety and coping skills of the relatives of children who stayed at hospital for spinal surgery, they found that family members with high levels of optimism used emotion-focused coping less and problem-focused coping more.

One of the basic needs of the human being is communication and people convey their feelings, and ideas through communication. Communication, used by the individual to lead his or her behaviors and fulfill his or her changing needs, is an integral part of his or her life and the base of socialization process. Communication skills make it possible to understand messages correctly in interpersonal relationships, and in the exchange of feelings and ideas. They also help to establish relationships. Messages which are conveyed through communication skills have the property of being one of the powerful tools in understanding others and helping them as well as its curing features on people (10).

The workspace of midwives and nurses includes events peculiar to all the stages of their lives, and it has results which are directly related to individuals’ health. It also does not leave any chance for mistake. It often requires taking quick and correct decisions, using effective communication skills and lastly experience and field knowledge. For this reason, nurses and midwives may face high levels of stress in their work. Unable to cope with stress, they may involve in collapse, psychosomatic disorders and depression. This can be reflected negatively on nursing, and can lead to financial loss and a variety of risks for patient’s health (11). Therefore, it is of great significance to promote positive thinking and communication skills to raise coping with stress (10, 12).

Tutuk et al. (13) found that as the class level of nursing students increases, their communication skills and predisposition to empathy improve. Age, being graduated from high school, attending social events, working or having worked have no effect on communication and predisposition to empathy scores. Lastly, students who stated to have problems in communication with their relatives were found to have low levels of communication and empathy skills. In a study in which communication skills of nursing and medical school students, working nurses and physicians were compared, it was determined that most working physicians, nurses and medical school students had more communication problems with patients whereas nursing students had less. It was thought that such a result was obtained because nursing students had training about interpersonal communication during vocational education, therefore they approached to this issue more sensitively (14).

One of the main goals of nursing and midwifery education is to provide students with effective communication skills, and to help them to reflect this on both work and personal lives. For this reason, there is a need to have enough data about students’ level of communication skills, and on what level life orientations affect communication skills, and how all these reflect on their coping attitudes. also there are not any studies to illustrate it.

## 2. Objectives

The present study was planed to investigate the associations between communication skills and life orientations of nursing and midwifery students (studying in universities in Istanbul) with coping attitudes with stress. This study tried to answer three questions:

What is the level of students’ life orientation, communication skills and coping attitudes?

Do students’ communication skills and life orientation have any correlation with coping with stress?

Is there any correlation between students’ communication skills and life orientation?

## 3. Materials and Methods

This is a cross sectional study. The study population consisted of 2572 nursing and midwifery students who were studying at seven universities in Istanbul in the academic year of spring 2009 - 2010. Through random selection, 1419 students participated in the study and completed the data collection tools. The aim of the research was first clarfied for students, and written informed consent was obtained from them. Then the tools were given to students. Aproximately, the students completed the questionaires in 20-25 minutes, and tools were withdrawn. To gather data, Personal Information Form for Students (PIFS), Life Orientation Test (LOT) and Communication Skills Scale (CSS) and Ways of Coping Inventory (WCI) were used.

Personal Information Form for Students (PIFS): It consisted of nine questions aimed to determine students’ age, sex, school of education, department, number of siblings, residency address, income, working condition, and attending social events.

Life Orieantation Test (LOT) is a scale with 12 items developed by Scheier and Carver (15) to determine the level of individuals optimism. The scale was adapted to Turkish by Aydın and Tezer (7) and its validity-reliability study was conducted. Correlation coefficient for frequency of life orientation test was 0.77. Scoring ranged from 0 to 4 in the scale, and it was in the following form: “Definitely disagree (0)”, “Disagree (1)”, “Doubtful (2)”, “Agree (3)” and “Definitely agree (4)”. The highest score is 31 and the lowest one is 0. Four items (2, 6, 7, and 10) were not included in the scoring. The other four items (3, 4, 12, and 14) shows pessimism level, and the rest of the items (7, 9, 10, and 15) optimism. A high score represents a high level of optimism (2,8,15).

Communication Skills Scale (CSS): It was first developed and used by Balcı and Ersanlı (16). Correlation coefficient for frequency of communication skills scale was 0.72. As a result of the factor analysis, the items were observed to be under three dimensions and taking these dimensions into consideration, they were called mental (0.83), emotional (0.73) and behavioral communication skills (0.82). There are 15 items that measure each dimension. The items are answered as “always (5)”, “usually (4), “sometimes (34)”, “rarely (2)”, “never (1)”. The highest score to get from the overall scale is 225 and the lowest is 45. The highest score to get from each subdimension of the scale is 75 and the lowest one is 15.

Ways of Coping Inventory (WCI): It was developed by Folkman and Lazarus (17) to determine what individuals do to cope with problems and stress in their lives. Contracted form of the scale was adapted to Turkish by Sahin and Durak (9), and its study of validity and reliability was performed. Correlation coefficient for frequency of coping strategies was 0.81. There are thirty items and five subdimensions. These subdimensions are secure approach to oneself, seeking social help, submissive approach, powerless/self-accusatory approach and optimistic approach. By adding subscores to these five subdimensions, coping strategy to be used is determined (2, 9, 17).

To conduct the study, necessary permissions were obtained from relevant institutions. Research aims were told to students and their approval was taken. It was reassured that none of the personal information gathered from the participant students would be disclosed to anybody, shared with anybody, and would be misused in a way that had been explained beforehand. The principle of loyalty and secrecy was followed strictly.

Statistical analyses were conducted using the Statistical Package for Social Sciences (SPSS Version 17.0 for Windows). Data analysis was performed through frequency, percentage, average, standard deviation, and Pearson Correlation test. P < 0.05 were considered statistically significant.

## 4. Results

The mean age was 21.01 ± 1.90 years. Also 92.4 % of the students were female, 55.3 % of them were studying at a public university, and 44.7 % at foundation universities. 74.6 % of them were studying nursing and 25.4 % midwifery. As for the family, 35.7 % of them had two siblings, and 38.3 % of them lived with their families. 81.9 % of them provided their own expenses and, 12.5 % of them worked in their free time, and 17.8 % of them were attending social activities (Table 1).

**Table 1.** Personal Characteristics of Students, (N=1419)

Characteristics	Number (%)
**Gender**	
Female	1311, (92.4)
Male	108, (7.6)
**Age Groups^a^, y**	
18 - 20	634, (44.7)
21 - 23	675, (47.6)
≥ 24	110, (7.7)
**School**	
Public University	635, (44.7)
Foundation University	784, (55.3)
**Department**	
Nursing	1058, (74.6)
Midwifery	361, (25.4)
**Siblings**	
One	79, (5.6)
Two	506, (35.7)
Three	415, (29.2)
Four and more	419, (29.5)
**Lived Places**	
With Family	543, (38.3)
Dormitory	423, (29.8)
With friend at home	304, (21.4)
Relative/Sibling with at home	66, (4.7)
Only at home	83, (5.8)
**Economic Status**	
Income an expense equal	1162, (81.9)
Income and expense not equal	257, (18.1)
**Occupied**	
Yes	178, (12.5)
No	1241, (87.5)
**Attending to Social Activities**	
Yes	252, (17.8)
No	1167, (82.2)

^a^ Average Age (Mean ± SD), 21.01 ± 1.90, (Min=18, Max=37)

It was determined that students’ life orientation lied in direction of optimism and their total score for communication skills scale (CSS), CSS subscales and coping with stress strategies respectively (Table 2). As for coping attitudes with stress, they were observed to adopt self confidence, helpless, optimistic approach, and seeking social help most and submissive approach at least (Table 2).

**Table 2.** Life Orientations, Communication Skills and Ways of Coping with Stress Scores of the Students, (N=1419)

	Min / Max	Mean ± SD
**Life Orientation Test (LOT)**	0.00 / 31.00	18.51 ± 4.54
**Communication Skills Score, Total (CSS)**	117.00 / 209.00	165.27 ± 15.39
**CSS, ** **subdimensions**		
Mental communication Skills	34.00 / 72.00	54.98 ± 5.58
Behavioral communication Skills	35.00 / 75.00	57.41 ± 6.03
Emotional communication Skills	29.00 / 69.00	52.88 ± 6.19
**Ways of Coping Inventory, subdimensions**		
Self-Confidence approach	0.00 / 21.00	13.61 ± 3.77
Optimistic approach	0.00 / 15.00	8.66 ± 2.72
Helpless approach	0.00 / 24.00	10.69 ± 4.12
Submissive approach	0.00 / 17.00	6.17 ± 3.13
Social help approach	0.00 / 12.00	7.57 ± 2.09

There was a positive correlation at medium level between their LOT scores and the scores for self confidence (r = 0.34, P < 0.001) optimistic (r = 0.42, P < 0.001), and seeking social help approaches (r = 0.17, P < 0.001) (Table 3).

**Table 3.** Correlation Between the Scores of Communication Skills and Life Orientation Test with Ways of Coping with Stress, (N=1419)

	Ways of Coping Inventory (WCI)					
	Self confidence Approach, r^a^	Optimistic Approach, r	Helpless Approach, r	Submissive Approach, r	Social Help Approach, r	P Value
**Life Orientation Test (LOT)**	0.34	0.42	-0.28	-0.15	0.17	0.001
**Total Communication Skills Score (CSS)**	0.46	0.37	-0.29	-0.36	0.29	0.001
**CSS, subdimensions**						
Mental communication Skills	0.36	0.31	-0.18	-0.24	0.21	0.001
Emotional communication Skills	0.43	0.33	-0.31	-0.36	0.31	0.001
Behavioral communication Skills	0.41	0.32	-0.26	-0.33	0.24	0.001

^a^ r; Correlation coefficient: 0. 0-0.24 not correlation- low level correlation, r: 0. 25-0.49 medium level correlation, r:0.50-0.74 strong level correlation, r: 0.75-1.00 most strongest level correlation

There was a significant positive correlation between students’ total score for CSS and scores for subdimensions of WCI, self confidence , optimistic and seeking social help approaches, and there was a significant negative correlation between CSS score and helplessand submissive approaches (Table 3).

When correlation between the average scores of CSS subdimensions and WCI subdimensions were analyzed, there was a significant positive correlation between the scores of mental communication skills and self confidence, 0.001 optimistic, and seeking social help approaches. There was a negative correlation between the scores of mental communication skills and helpless and submissive approaches (Table 3).

There was a positive correlation between the scores of their emotional communication skills and self confidence , optimistic and seeking social help approaches, and a negative correlation between the scores of emotional communication skills and helpless and submissive approaches (Table 3).

Also, there was a positive correlation between the scores of behavioral communication skills and self confidence,optimistic, and seeking social help approaches. There was a negative correlation between the scores of behavioral communication skills and helpless and submissive approaches (Table 3).

**Figure 1.**
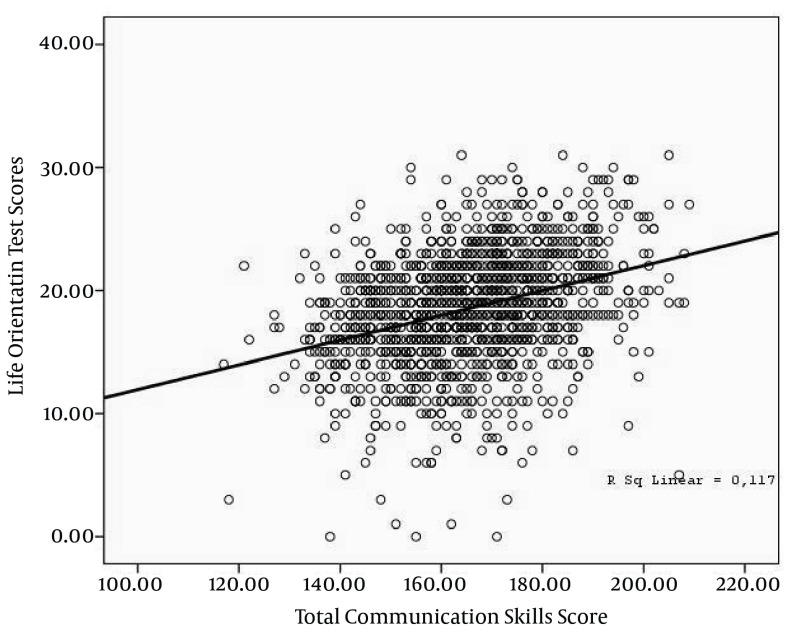
Correlation Between the Scores of Communication Skills of Students and Life Orientation Test Scores, (N=1419)

A positive correlation between students’ total scores for CSS and LOT scale average score was found. There was direct correlation between the scores of LOT and all subdimensions of CSS (Table 4) and (Figure 1).

**Table 4.** Correlation Between Communication Skills and Life Orientation Test Scores (N=1419)

	Life Orientation Test (LOT), r^I^	P Value
**Total Communication Skills Score (CSS)**	0.34	0.001
**Subdimensions**		
Mental communication Skills	0.23	0.001
Behavioral communication Skills	0.31	0.001
Emotional communication Skills	0.34	0.001

^I^ Pearson correlation test , r: 0. 0-0.24 not correlation- low level correlation, r: 0. 25-0.49 medium level correlation ilişki, r: 0.50-0.74 strong level correlation, r: 0.75-1.00 most strongest level correlation

## 5. Discussion

According to the results of the study, most of the students were female, nursing students and studying in a public university. Students’ life orientation lie in direction of optimism and communication skills was at medium level. Despite the students’ had used different coping strategies in stressful situations, most strategies were submissive and self confidence approaches.

Nursing and midwifery students might face problems such as remaining separate from the family, making new friends, fear of being alone, financial difficulties, not being able to adapt to life in dormitory, not finding what they had expected, not realizing their plans, and not internalizing the department when they first start their education in university (18, 19). Such negative feelings and stressful life students experience can lead to various mental problems, decrease in academic success, and emotions such as hopelessness for the future and desperateness (4, 20). Nevertheless, the researches show that individuals, who use of effective communication skills and adopt optimistic life orientation, cope with stress more effectively, and protect their physical and mental health better (21, 22). According to the findings of the study, it was found that students’ life orientations lie in optimistic direction and on average.

Students’ total CSS score was found to be higher according to the findings of Korkut (23), Tutuk et al. (13), Arifoğlu and Razı (24) and Razı et al. (25) studies. It was found that students used behavioral, mental and emotional communication skills out of CSS subdimensions. In Tutuk’s (13) and Akyurt’s (26) study, it was also seen that students rather used mental, behavioral and emotional communication skills.This result gave rise to thought that students could transfer what they learnt through their education into practice when the necessity of communication skills are taken into consideration in nursing and midwifery which are based upon understanding the individuals and helping them.

As for coping strategies with stress, students were observed to use secure approach to oneself, powerless/self-accusatory, optimistic,and seeking social help approaches more respectively and submissive approach the least. In some other studies, however, emotion-focused and problem-focused coping attitudes are seen to be used in coping with stress (9, 11). As advised in the literature, students’ using these two coping attitudes together was considered as a positive and expected result.

When the correlation between LOT and WCI scores is considered, as life orientation scores increase, their scores for secure approach to oneself, optimistic and seeking social help approach increase while scores for powerless/self-accusatory and submissive approach decrease. When similar studies are analyzed; Nes and Segerstrom (27) pinpointed that there is a high correlation between optimistic life orientation and coping with stress; and also students with high levels of optimism can cope with health problems more effectively. Study findings support previous studies and gave rise to thought that students with optimistic life orientation trust themselves more, are more bellicose and could ask for help from their families or friends in case of a problem and cope with stress more effectively (7, 8).

It was also observed that as students’ total CSS score and mental, behavioral, and emotional communication skills subdimension scores increase, scores for secure approach to oneself, optimistic, and seeking social help approaches increase. However, scores for powerless/self-accusatory and submissive approaches decrease. It is stated in the literature that individuals with effective communication skills are those who can deal with problems more effectively, are more self-confident, behave in an objective manner in the face of problems and think problem/solution-based, are happy and productive and who have good relationships with others. On the other hand, people who are not successful in interpersonal relationships, prove to be incompetent in dealing with problems; trust themselves and others less and who feel anxiety at great levels. Study findings are parallel to those which were conducted before and they set us thinking that individuals with high levels of communication skills could express themselves more comfortably, cope with stress more effectively, would have positive expectations from the future and would be more sensitive to the problems of healthy/sick people in their work life which requires team collaboration (7, 25).

To be happy and cheerful, the individual first need to evaluate himself or herself and spread this approach to his or her surroundings. Such behavior style would help to have better results both in the individual and his or her social relations (7, 11).

When literature is analyzed, only a few studies which focus on correlation between individuals’ communication skills and optimistic life orientations could be found. Bozkurt (28) analyzed personalities of adults who established successful communication with others, and stated that they are individuals who are self-confident and mature emotionally and intellectually; thereby individuals with strong communication skills are happy and their life orientation would develop in a positive direction.

It was seen in this study that there is a positive correlation at a medium level between students’ CSS total score and LOT scale average score; a low correlation between their LOT scale score averages and mental communication skills subdimension scores; a positive correlation at a medium level between behavioral and emotional communication skills subdimension scores. These findings showed that students who adopt an optimistic approach towards life can have a better level of communication skills.

Students’ communication skills and life orientation scores were not at a desirable level. However, it is favorable that students preferred self confidence approach most as coping with stress attitude. Accorrding to these results, the following suggestions can be made: including courses in nursing curriculum which can improve their communication skills, including training programme which can increase students’ optimistic life orientations, teaching them effective coping attitudes with stress, organizing counseling services to help students with social and psychological problems, enhancing existing counseling services, increasing the number of events which can bring different department together within the university, supporting students to attend to social and cultural activities.
